# Self-reported psychopathy in the Middle East: a cross-national comparison across Egypt, Saudi Arabia, and the United States

**DOI:** 10.1186/s40359-015-0095-y

**Published:** 2015-10-29

**Authors:** Robert D. Latzman, Ahmed M. Megraya, Lisa K. Hecht, Joshua D. Miller, D. Anne Winiarski, Scott O. Lilienfeld

**Affiliations:** Department of Psychology, Georgia State University, PO Box 5010, Atlanta, GA 30302-5010 USA; Department of Psychological Sciences, Qatar University, Doha, Qatar; Department of Psychology, University of Georgia, Athens, GA USA; Department of Psychology, Emory University, Atlanta, GA USA

**Keywords:** Psychopathy, Cross-national, Cross-cultural, Middle East, Five factor model personality

## Abstract

**Background:**

The construct of psychopathy is sparsely researched in the non-Western world, particularly in the Middle East. As such, the extent to which the psychopathy construct can be generalized to other cultures, including Middle Eastern Arab cultures, is largely unknown.

**Methods:**

The present study investigated the cross-cultural/national comparability of self-reported psychopathy in the United States (*N* = 786), Egypt (*N* = 296), and Saudi Arabia (*N* = 341).

**Results:**

A widely used psychopathy questionnaire demonstrated largely similar properties across the American and Middle Eastern samples and associations between Five Factor Model (FFM) personality and psychopathy were broadly consistent. Nevertheless, several notable cross-cultural differences emerged, particularly with regard to the internal consistencies of psychopathy dimensions and the correlates of Coldheartedness. Additionally, in contrast to most findings in Western cultures, associations between psychopathy and FFM personality varied consistently by gender in the Egyptian sample.

**Conclusions:**

These findings lend preliminary support to the construct validity of self-reported psychopathy in Arabic-speaking cultures, providing provisional evidence for the cross-cultural generalizability of certain core characteristics of psychopathy.

## Background

The construct of psychopathy has long been controversial, owing to a host of unanswered questions regarding its boundaries, assessment, and etiology [31]. One key unresolved issue concerns potential cross-cultural differences in the phenotypic manifestations and external correlates of psychopathy. This question bears important implications for understanding the extent to which the psychopathy construct can be generalized to other cultures, including non-Western cultures. As a number of scholars [41, 45] have observed, investigations of differences in psychopathy are limited largely to comparisons of (a) Whites versus African-Americans and (b) American, European, and Australian samples. Moreover, the findings from such studies are mixed. Some investigators have reported somewhat lower validity for psychopathy dimensions (especially those tied to impulsive and antisocial traits) in predicting laboratory performance and aggression among African-Americans than Whites [4, 11, 16, 42], whereas others have reported few or no differences [17, 44]. Cross-national investigations of psychopathy are even more sparse and difficult to interpret [46].

The goal of the current study was to examine the construct validity of a self-reported psychopathy measure among undergraduate students in three nations, one Western (i.e., U.S.) and two Middle Eastern (i.e., Saudi Arabia, Egypt), by testing the inter-correlations among the Psychopathic Personality Inventory-Revised (PPI-R) [19] subscales and factors as well as their associations with Five Factor Model (FFM) personality traits. Such work is important for ascertaining the extent to which the psychopathy construct is meaningful in cultures that are markedly different from those in North America and Europe, where the vast majority of psychopathy research has been conducted.

### Psychopathy in the Middle East

The generalizability of psychopathy across cultures is of interest for research and clinical purposes, as researchers have questioned the extent to which personality pathology is culture-bound [8, 28, 41]. Nevertheless, although a few researchers have examined scores on the MMPI or MMPI-2 in Arabic-speaking non-Western samples [3], this research has not focused on psychopathic or antisocial traits per se. Additionally, published studies that have examined measures of psychopathy-related traits, such as the MMPI or MMPI-2 Psychopathic deviate scale, have largely described approaches to translation and item-discrimination characteristics rather than external correlates [40]. More broadly, only a handful of studies has examined psychopathy in non-Western cultures. Most of these investigations have used the Psychopathy Checklist-Revised (PCL-R; [13]) or its various iterations. For example, utilizing a short version of the PCL-R among Iranian prisoners, Shariat et al. [39] found a similar factor structure to the original PCL-R standardization sample, although some culture-specific findings emerged. Specifically, items loaded differently on higher-order factors in each sample. Although no consistent pattern of higher or lower loading items emerged across samples, most of the significant differences were related to content associated with an arrogant and deceitful interpersonal style and deficient emotional experience (i.e., PCL-R Factor 1). Recent studies have also examined the correlates of psychopathy using self-report measures of personality, although most of these investigations have focused on Western samples [41].

We located only one published study examining psychopathy and personality in a non-Western sample. Using a self-report version of the PCL-R, Ghaderi et al. [12] examined associations between psychopathy and FFM personality among incarcerated Iranian prisoners. When PCL-R subdimensions were examined, both factors were strongly negatively associated with Agreeableness and moderately negatively associated with Conscientiousness and Openness. Corroborating findings in Western cultures [10, 22], results from multivariate analyses statistically predicting a global psychopathy score revealed significant main effects for low Agreeableness, most strongly, and low Conscientiousness [12]. In a broader investigation, Neumann and colleagues [30] reported on psychopathic traits assessed using the Self-Report Psychopathy (SRP) [32] scale, a questionnaire designed to be consistent with a PCL-R conceptualization of psychopathy, within a large world-wide sample. Respondents from the Middle East reported higher scores on the interpersonal facet, with more moderate scores across the other three facets, which assess affective, lifestyle, and antisocial aspects of psychopathy. Although Neumann et al. [30] included a Middle East region in their analyses, questionnaires were not administered in Arabic in any of the countries included in this region (i.e., Israel, Lebanon, Turkey), nor were Egypt and Saudi Arabia surveyed (sample information detailed in [38].

Taken together, this small literature suggests the emergence of a similar, although perhaps not identical, psychopathy construct in Middle Eastern samples. Nonetheless, the extent to which the core features of psychopathy are generalizable to cultures whose enculturation and socialization differ from those of Western culture remains unclear.

Underscoring the need for cross-cultural examinations of psychopathy is the recently published Diagnostic and Statistical Manual of Mental Disorders, 5th Edition (*DSM-5*; [2]. Similar to previous editions, *DSM-5* notes that signs and symptoms must “deviate markedly from the expectations of the individual’s culture” ([2], p. 645) to be regarded as pathological, highlighting the need for cultural sensitivity and awareness in the cross-cultural assessment and diagnosis of mental disorders. Personality disorders such as psychopathy are commonly believed to be among the most culturally-dependent diagnoses [8], rendering the need for cross-cultural/national investigations of conditions such as psychopathy even more vital [41].

### Self-Reported Psychopathy and FFM Personality

Psychopathy has repeatedly been found to be a multidimensional construct [31], and a number of measures have been developed for assessing its sub-dimensions. Although questionnaire indices of psychopathy have often been viewed with skepticism [18], growing evidence attests to their validity in non-incarcerated populations. Specifically, self-report psychopathy scores demonstrate substantial convergence with informant reports [27] and there is little evidence to suggest that psychopathic individuals in non-forensic settings are especially prone to positive impression management, at least in non-incentivized research contexts [34]. One of the most widely-used, well-validated self-report measures of psychopathy designed to detect the personality features of the condition is the PPI-R, which consists of eight content scales that often load onto two factors, Fearless Dominance (FD) and Self Centered Impulsivity (SCI), with the exception of Coldheartedness, which does not load highly on either factor [6] (c.f. [29]) for an alternative factor structure). FD is associated with many of the core affective and interpersonal features of psychopathy, including social and physical boldness, charm, glibness, and relative immunity to anxiety, and is tied to the largely socially adaptive features of psychopathy [20] (c.f. [26]), for criticisms of the FD construct). In contrast to FD, SCI is associated with most of the behavioral features of psychopathy, including manipulativeness, egocentricity, aggressiveness, impulsivity, and antisociality [5, 6]. Coldheartedness reflects a separate subgroup of traits, potentially tied to other affective features of psychopathy, such as lack of guilt and remorse, callousness, and absence of social emotions.

The PPI-R lends itself well to cross-cultural studies of psychopathy given that as it was designed to (a) minimize the use of culture-specific idioms and (b) present items at a relatively low reading level that are applicable to a broad range of samples. In addition, in contrast to other questionnaire measures of psychopathy, the PPI-R has been officially translated and back-translated into Arabic (PAR, http://www4.parinc.com/Products/PermsLicensing.aspx?id=23). In these respects, it may be well suited to cross-cultural studies, including those in the Middle East.

Several investigators have examined associations between PPI-R higher-order factors and FFM personality traits. Summarizing these findings, recent meta-analytic results reported that FD was related to Neuroticism (*r* = −.50) and Extraversion (*r* = .50), with a smaller contribution from Openness (*r* = .25); the associations with Agreeableness and Conscientiousness were negligible. In contrast, SCI reflected contributions from Agreeableness (*r* = −.49) and Conscientiousness (*r* = −.51), with a lesser contribution from Neuroticism (*r* = .30); the associations with Extraversion and Openness were negligible [26]. Coldheartedness, however, appears to be somewhat more difficult to capture with FFM domains [35], although a combination of low Agreeableness and low Conscientiousness explained the most variance in this dimension. Taken together, the PPI factors evidence differential associations with FFM traits.

With some exceptions, the FFM appears to be reasonably robust across cultures [23, 24]. Nevertheless, the FFM trait of Openness has been less consistent in its manifestation, in some cases not emerging clearly in certain cultures (e.g., [37]). The reason for these findings are unclear, although the Openness trait may be less relevant in more traditional cultures in which behavioral options, such as the ability to pursue one’s artistic, musical, or intellectual interests, are constrained [33]. Interestingly, in the original factor analyses identifying the FFM, Tupes and Christal [43]) dubbed this dimension “Culture,” underscoring the extent to which it assessed cultural interests. These interests, and the extent to which people are able to actualize them, may in turn differ across cultures.

### Current study

We aimed to examine the cross-cultural/national comparability of psychopathy in a cultural group that has received no known systematic investigation in this regard, namely, individuals in Middle Eastern countries. Specifically, we examined the psychometric properties of self-reported psychopathy scores among undergraduate students in two large Arabic-speaking countries - Egypt and Saudi Arabia - and compared them with scores derived from a large undergraduate American sample. We also examined FFM personality correlates of psychopathy in the American and Egyptian samples.

Although this is the first study of these questions in Middle Eastern Arabic-speaking samples, we advanced a number of provisional *a priori* hypotheses deduced from the broader cross-cultural literature. First, with regard to associations with FFM personality domains, consistent with the extant literature on Western samples, we expected SCI (and its component traits) and Coldheartedness to be most strongly negatively associated with Agreeableness and Conscientiousness. In contrast, we expected FD (and its component traits) to be most strongly positively associated with Extraversion and negatively correlated with Neuroticism.

Finally, although broadly comparable correlates of psychopathy across genders have generally emerged in Western samples [7, 27], little is known regarding the role of gender in non-Western (i.e., Middle-Eastern) samples. Thus, in a set of more exploratory analyses, we also examined the potential moderating role of gender in the association between psychopathy and FFM personality domains. These analyses are important given that male and female gender roles and expectations tend to be much more clearly demarcated in most Arabic than in Western cultures.

## Methods

### Participants

#### United States

American participants were 786 undergraduates (*M*_age_ = 19.34, *SD* = 2.19; 53.1 % male) who completed questionnaires in partial fulfillment of a research exposure requirement at a large public university in the Southeastern United States. Most participants identified as White (83.1 %), with the remainder identifying as Asian (6.6 %), Black/African-American (6.2 %), or other (4.1 %). Written informed consent for participation in the study was obtained from all participants and all procedures were approved by the University of Georgia’s Institutional Review Board.

#### Egypt

Egyptian participants were 296 undergraduates (*M*_age_ = 18.56, *SD* = 0.86; 33.1 % male) who volunteered to completed questionnaires in a group setting at a large public university in Egypt. Written informed consent for participation was obtained from all participants and the Department of Psychology, Faculty of Arts, Menoufia University approved the ethical compliance for the use of human subjects according to the Helsinki Declaration. Participants were not compensated for completing the measures.

#### Saudi Arabia

Saudi Arabian participants were 341 undergraduates (*M*_age_ = 18.36, *SD* = 0.74; 56.6 % male) who completed questionnaires as volunteers at large public university in a metropolitan city in Saudi Arabia. Written informed consent for participation was obtained from all participants and the University College of Qunfudah, Umm Al-Qura University approved the ethical compliance for the use of human subjects according to the Helsinki Declaration. Similar to the Egyptian sample, volunteers were administered all study materials in group settings and were not compensated for their participation.

### Measures

#### Psychopathic Personality Inventory-Revised

The Psychopathic Personality Inventory-Revised (PPI-R; [19] is a 154-item self-report measure of psychopathy in which respondents describe themselves using a 4-point Likert-type scale. The PPI-R yields a total score reflecting global psychopathy, as well as scores on eight content scales, reflecting lower-order features of psychopathy. Higher-order factor analyses of these scales have sometimes yielded a two factor solution [6], with FD consisting of summed scores on Fearlessness, Social Potency, and Social Immunity content scales and SCI consisting of summed scores on Machiavellian Egocentricity, Rebellious Nonconformity, Blame Externalization, and Carefree Nonplanfulness content scales. As noted earlier, Coldheartedness is typically treated as a stand-alone factor in analyses. In addition, the PPI-R contains several validity scales, including Deviant Responding (DR) and Inconsistent Responding (INC), with the latter especially relevant for the evaluation of translated versions. The DR scale consists of bizarre items that are unlikely to be endorsed by individuals with genuine psychopathology, whereas the INC scale consists of moderately to highly correlated item pairs, with inconsistent responding within pairs generating higher scores. The vast majority of participants across samples (>98 %) fell well within traditional cut-scores [19] for the assessment of valid responding on both measures. Egyptian and Saudi Arabian participants completed the officially translated Arabic version, whereas American participants completed the English version.

#### NEO-Five Factor Inventory

The NEO-Five Factor Inventory (NEO-FFI; [9]) is a 60-item self-report measure designed to assess the FFM domains using a 5-point Likert-type scale: Neuroticism, Extraversion, Openness, Agreeableness, and Conscientiousness. NEO-FFI items were translated from English into Arabic by a bilingual scientist familiar with the FFM (A.M.M.). Items were back-translated by an Arabic scholar fluent in both Arabic and English with extensive experience providing translational services to publishers. The back-translated items were subsequently independently evaluated by three of the current authors (i.e., R.D.L., J.D.M., S.O.L.). Psychometric properties including item-total correlations and internal consistencies were examined in an iterative manner after removing items deemed potentially questionable. None of the revisions led to discernible improvement in the psychometric properties of scales. Egyptian participants completed the translated Arabic version, whereas American participants completed the English version. As a result of time constraints when these data were collected, Saudi Arabian participants did not complete the NEO-FFI.

### Translations

Both the PPI-R and the NEO-FFI were translated into simple modern formal “standard” Arabic by Egyptian scholars. This form of Arabic is understandable across all Arabic- speaking countries as it is the same across all countries. Importantly, although slang terms do vary slightly across Arabic speaking countries, they are widely understood.

### Analyses

We first examined the internal consistencies (Cronbach’s alphas) of the psychopathy and FFM scales across groups. We then examined zero-order correlations among the eight PPI-R scales across groups. To examine unique associations between psychopathy and FFM traits in the Egyptian and American samples, we used multivariate linear regression models. Although studies in Western samples have found the correlates of psychopathy to be broadly similar across genders [7, 27], the potential moderating effects of gender in non-Western samples is largely unknown. As such, gender was included in the first step of our regression analyses, and gender by FFM trait interactions were included in the third and final step.

Although our primary analyses focused on the external correlates of psychopathy measures across cultures, we also conducted secondary analyses examining the factorial validity of the PPI-R factors. Specifically, as an initial step toward examining the possibility of differing factor structures across samples, we fit multi-group confirmatory factor analysis (CFA) models for FD and SCI to test the measurement equivalence of psychopathy subdimensions across the three samples. A model in which loadings were constrained (i.e., factorial invariance) across the three groups was compared with a model in which loadings were freed (i.e., factorial variance) to vary across groups. PPI-R subscales were used as indicators for FD and SCI. As the Coldheartedness scale is the only indicator of its eponymous factor, it was excluded from these analyses. Model fit was evaluated using the Bayesian information criterion (BIC) and Akaike Information Criteria (AIC). These approaches to model selection involve the comparison of omnibus criteria (i.e., BIC; AIC) that prioritize a model’s goodness of fit and reward more parsimonious models, although AIC penalizes the number of parameters less strongly than does BIC [1, 36].

## Results

### Preliminary analyses

Internal consistency values of the PPI-R scales are shown in Table 1. Although reliabilities were good to excellent in the American sample, reliabilities evidenced substantial variability across scales in the Egyptian and Saudi Arabian samples, ranging from adequate to problematic. Most notably, for the PPI-R scales, internal consistencies were equal to or below .60 in both Arabic samples for Stress Immunity and Fearlessness. The internal consistency of the Social Influence scale in the Egyptian sample was well below acceptable levels (alpha = .45) and internal consistencies of the Carefree Nonplanfulness and Stress Immunity scales were below .60 in the Saudi Arabian sample. Although internal consistencies were adequate for NEO-FFI scales in the American sample, modest, in one case, extremely low, internal consistency values emerged for the NEO-FFI in the Egyptian sample (*Mdn* alpha = .53), with that of Openness close to zero (alpha = .09).

**Table 1:** Internal Consistencies (Cronbach’s alphas)

	α		
	US	Egypt	Saudi Arabia
Psychopathic Personality Inventory-Revised (PPI-R)			
Machiavellian Egocentricity (ME)	.84	.72	.63
Rebellious Nonconformity (RN)	.85	.69	.60
Blame Externalization (BE)	.86	.72	.62
Carefree Nonplanfulness (CN)	.88	.65	.59
Social Influence (SOI)	.85	.45	.62
Fearlessness (F)	.87	.55	.60
Stress Immunity (STI)	.83	.51	.56
Coldheartedness (C)	.86	.72	.65
NEO-Five Factor Inventory (NEO-FFI)			
Neuroticism	.86	.53	
Extraversion	.80	.59	
Openness	.71	.09	
Agreeableness	.81	.44	
Conscientiousness	.86	.64	

*Note.* US *N* = 786. Egypt *N* = 296. Saudi Arabia *N* = 341. As described in the text, NEO-FFI data was not available on the Saudi Arabian sample

Interrelations among PPI-R subscales and factors are displayed in Tables 2, 3, 4. Interrelations among subscales were relatively consistent across samples, although PPI-R Blame Externalization was a notable exception. Specifically, Blame Externalization evidenced smaller associations with the other subscales in the Saudi Arabian sample compared to the Egyptian and American samples, which were much more similar. With regard to factor scores, groups again evidenced similar bivariate associations. The notable exception was Coldheartedness, which evidenced stronger associations with the PPI-R subscales and other factors scores in the American sample compared to the two Arabic samples.

**Table 2:** Interrelations among Psychopathic Personality Inventory-Revised Scales and Factors: US Sample

PPI-R scale/factor	Total	ME	RN	BE	CN	SOI	F	STI	SCI	FD	C
Scale											
Total											
Machiavellian Egocentricity (ME)	.72										
Rebellious Nonconformity (RN)	.73	.51									
Blame Externalization (BE)	.50	.50	.39								
Carefree Nonplanfulness (CN)	.65	.41	.49	.46							
Social Influence (SOI)	.33	.14	.11	-.17	-.15						
Fearlessness (F)	.65	.29	.50	.08	.21	.27					
Stress Immunity (STI)	.27	-.11	-.00	-.32	-.07	.29	.33				
Factor											
Self-Centered Impulsivity (SCI)	.85	.79	.77	.75	.78	-.02	.35	-.16			
Fearless Dominance (FD)	.59	.17	.31	-.16	.01	.73	.76	.69	.11		
Coldheartedness (C)	.66	.47	.26	.51	.51	.02	.22	.25	.49	.22	
Mean	290.76	42.99	32.68	30.27	37.83	47.01	33.31	33.89	143.76	114.22	32.77
Standard Deviation	34.79	8.16	7.51	7.02	8.40	7.77	8.26	6.26	24.01	16.26	7.42

*Note. N* = 786. 53.1 % Male. PPI-R = Psychopathic Personality Inventory-Revised

**Table 3:** Interrelations among Psychopathic Personality Inventory-Revised Scales and Factors: Egyptian Sample

PPI-R scale/factor	Total	ME	RN	BE	CN	SOI	F	STI	SCI	FD	C
Scale											
Total											
Machiavellian Egocentricity (ME)	.80										
Rebellious Nonconformity (RN)	.75	.66									
Blame Externalization (BE)	.68	.63	.78								
Carefree Nonplanfulness (CN)	.39	.39	.14	.22							
Social Influence (SOI)	.27	.14	-.01	.00	-.14						
Fearlessness (F)	.40	.26	.45	.11	-.17	.07					
Stress Immunity (STI)	.10	-.20	-.10	-.16	-.12	.23	.01				
Factor											
Self-Centered Impulsivity (SCI)	.90	.86	.80	.80	.46	.02	.24	-.20			
Fearless Dominance (FD)	.42	.12	.21	-.01	-.22	.67	.62	.31	.05		
Coldheartedness (C)	.40	.20	.05	.15	.46	-.09	-.22	.07	.27	-.14	
Mean	303.99	50.39	38.10	39.93	34.57	44.49	36.37	30.93	162.99	111.78	29.22
Standard Deviation	26.78	8.55	7.60	6.94	6.33	5.69	6.32	5.25	21.81	10.92	6.35

*Note. N* = 296. 33.1 % Male. PPI-R = Psychopathic Personality Inventory-Revised

**Table 4:** Interrelations among Psychopathic Personality Inventory-Revised Scales and Factors: Saudi Arabian Sample

PPI-R scale/factor	Total	ME	RN	BE	CN	SOI	F	STI	SCI	FD	C
Scale											
Total											
Machiavellian Egocentricity (ME)	.64										
Rebellious Nonconformity (RN)	.73	.51									
Blame Externalization (BE)	.45	.37	.37								
Carefree Nonplanfulness (CN)	.34	.02	.14	.10							
Social Influence (SOI)	.34	.07	.12	-.21	-.18						
Fearlessness (F)	.54	.31	.40	.20	-.09	.09					
Stress Immunity (STI)	.13	-.22	-.13	-.33	-.12	.31	.07				
Factor											
Self-Centered Impulsivity (SCI)	.82	.74	.77	.68	.46	-.06	.32	-.30			
Fearless Dominance (FD)	.53	.11	.22	-.14	-.20	.73	.62	.63	.01		
Coldheartedness (C)	.39	.07	.09	.01	.37	-.00	-.10	.10	.19	-.02	
Mean	297.13	49.19	34.69	36.72	38.44	45.27	33.98	30.28	159.04	109.53	28.56
Standard Deviation	22.74	7.21	6.47	6.17	6.24	6.55	6.49	5.14	17.38	12.04	5.59

*Note. N* = 341. 56.6 % Male. PPI-R = Psychopathic Personality Inventory-Revised

To examine the similarity of the interrelations among the PPI-R scales and factors across groups, we calculated double-entry q-correlations [23] among the correlations presented in Tables 2 to 4, which measure the absolute similarities of these correlations. These intraclass correlations ranged from .81 (Egypt–US) to .91 (Egypt–Saudi Arabia) with a median of .82. We conducted similar analyses using the correlations manifested by the three major PPI-R factors with the seven subscales and PPI-R total score. Although the patterns of inter-relations were similar for FD across groups (Saudi Arabia-Egypt: *r*_*ICC*_ = .92; Saudi Arabia–US: *r*_*ICC*_ *=* .96; Egypt–U.S.: *r*_*ICC*_ = .82) and SCI (Saudi Arabia-Egypt: *r*_*ICC*_ = .98; Saudi Arabia–US: *r*_*ICC*_ *=* .94; Egypt–U.S.: *r*_*ICC*_ = .95), they diverged quite substantially for Coldheartedness, particularly between the U.S. and Middle Eastern samples (Saudi Arabia-Egypt: *r*_*ICC*_ = .88; Saudi Arabia–US: *r*_*ICC*_ *=* .14; Egypt –U.S.: *r*_*ICC*_ = .38), suggesting that this PPI-R dimension functions differently across these nations.

### Cross-cultural associations between FFM personality and psychopathy

Due to the problematic internal consistency of Openness in the Egyptian sample, it was excluded from subsequent analyses. Interrelations among FFM scales within samples are shown in Table 5; the pattern of intercorrelations among the FFM domains was nearly identical across the two samples (*r*_*ICC*_ = .96). Because of the relatively low internal consistencies of some scales, particularly in the Egyptian sample, all correlations were corrected for attenuation (using Cronbach’s alphas as indices of reliability). Disattenuated correlations between PPI-R scales and personality are shown in Table 6.

**Table 5:** Interrelations among NEO-Five Factor Inventory Scales: US and Egyptian samples

	Neuroticism	Extraversion	Agreeableness	Conscientiousness
Neuroticism	--	-.42	-.31	-.32
Extraversion	-.40	--	.51	.51
Agreeableness	-.24	.39	--	.48
Conscientiousness	-.36	.31	.34	--

*Note.* US sample (*N* = 786) is on the below the diagonal and Egyptian sample (*N* = 288) is above the diagonal. All correlations significant *p* < .001. As described in the text, Openness was not included in analyses

**Table 6:** Disattenuated bivariate correlations between Psychopathic Personality Inventory-Revised scales and factors and Five Factor Model: US and Egyptian samples

PPI-R scale/factor	N	E	A	C
Scale				
Machiavellian Egocentricity (ME)	.19/.24	-.15/-.23	-.75/-.76	**-.39/-.04**
Rebellious Nonconformity (RN)	.14/.12	**-.14/-.42**	**-.51/-.86**	**-.47/-.31**
Blame Externalization (BE)	.55/.47	**-.32/-.51**	**-.59/-.79**	**-.40/-.24**
Carefree Nonplanfulness (CN)	.30/.41	**-.26/-.65**	-.48/-.59	-.76/-.87
Social Influence (SOI)	**-.48/-.66**	**.65/.45**	**.03/.26**	.21/.31
Fearlessness (F)	-.24/-.20	.18/.17	-.24/-.19	-.20/-.17
Stress Immunity (STI)	**-.82/-.69**	*.23/.06*	.08/.00	.15/.13
Self-Centered Impulsivity (SCI)	.36/.36	**-.27**/**-.52**	**-.72**/**-.92**	**-.64**/**-.40**
Fearless Dominance (FD)	-.67/-.73	**.49**/**.33**	-.08/.01	.05/.11
Coldheartedness (C)	**-.17/.21**	**-.30/-.63**	-.66/-.70	-.26/-.37

*Note.* US sample (*N* = 786) is on the left and Egyptian sample (*N* = 288) is on the right. Correlations in *italics* are significantly different from one another (Fisher’s z-test) at *p* < .05. Correlations in **boldface** are significantly different from one another (Fisher’s z-test) at *p* < .01

As described in the text, Openness was not included in analyses

PPI-R = Psychopathic Personality Inventory-Revised

Across both samples, Machiavellian Egocentricity was most strongly associated with low Agreeableness and also evidenced a significant negative association with Conscientiousness in the American sample, with this correlation approaching zero in the Egyptian sample. Although the absolute magnitude of association was greater in the Egyptian sample, Rebellious Nonconformity was associated most strongly with low Agreeableness in both samples, followed by low Conscientiousness in the American sample. For Egyptians, however, Rebellious Nonconformity evidenced its second strongest association with low Extraversion followed by low Conscientiousness. Blame Externalization was most strongly associated with low Agreeableness but, again, the magnitude of this association was greater in the Egyptian sample. Blame Externalization also evidenced significant associations with Neuroticism and low Conscientiousness across samples. Carefree Nonplanfulness was most strongly associated with low Conscientiousness in both samples, followed by low Agreeableness in the American sample and low Extraversion in the Egyptian sample. Although associated most strongly with low Neuroticism and Extraversion in both samples, Social Influence was most strongly correlated with Extraversion in the American sample, whereas the strongest association was with low Neuroticism in the Egyptian sample. Fearlessness evidenced a similar pattern of correlations in both samples, with weak associations with Extraversion and low Neuroticism, low Agreeableness, and low Conscientiousness. Although the magnitude of the association was greater in the American sample, Stress Immunity was strongly associated with low Neuroticism in both samples. Lastly, Coldheartedness was most strongly correlated with low Agreeableness followed by low Extraversion in both samples, although the magnitude of the latter association was significantly larger in the Egyptian sample; in addition, the relations between Coldheartedness and Neuroticism changed direction across the samples (U.S.: negative; Egypt: positive). With regard to correlations between FFM domains and SCI and FD PPI-R factor scores, the general pattern of associations was broadly consistent across samples. In contrast, whereas Coldheartedness was weakly negatively associated with Neuroticism in the American sample, this association was weakly positive in the Egyptian sample.

### Predicting psychopathy scores using the FFM domains

Results of multivariate regression analyses predicting each of the PPI-R scales and factors controlling for gender in the American and Egyptian samples are presented in Table 7. In Step 1 in the American sample, with the exception of Blame Externalization and Social Influence, gender evidenced significant associations with all PPI-R scales and factors, with males reporting higher scores across all scales. Gender accounted for less variance in scores in the Egyptian sample, evidencing significant associations with only Carefree Nonplanfulness, Stress Immunity, Coldheartedness, and the PPI-R Total score; men again reported higher levels than women.

**Table 7:** Multivariate Regression Analyses Predicting Psychopathic Personality Inventory-Revised Scales and Factors from the Five Factor Model: US & Egyptian Sample

Total	ME	RN	BE	CN	SOI	F	STI	SCI	FD	C	
*β*	*β*	*β*	*β*	*β*	*β*	*β*	*β*	*β*	*β*	*β*	
US Sample: *N =* 786											
**Step 1: Gender**	-.26*	-.16*	-.13*	-.07	-.10*	.06	-.27*	-.28*	-.15*	-.22*	-.27*
**Step 2: FFM Traits**											
Δ*R* ^*2*^	.46^a^	.40^a^	.25^a^	.38^a^	.46^a^	.37^a^	.16^a^	.46^a^	.53^a^	.43^a^	.35^a^
Neuroticism	-.24*	.05	-.02	.38*	.01	-.27*	-.22*	-.73*	.12*	-.52*	-.36*
Extraversion	.24*	.19*	.13*	.07	.06	.51*	.26*	-.04	.14*	.36*	-.14*
Agreeableness	-.58*	-.62*	-.36*	-.39*	-.22*	-.25*	-.23*	-.02	-.51*	-.24*	-.52*
Conscientiousness	-.37*	-.16*	-.32*	-.09*	-.60*	-.00	-.24*	-.10*	-.39*	-.16*	-.12*
Egyptian Sample: *N* = 288											
**Step 1: Gender**	-.18**	-.04	.01	.01	-.21**	.05	-.10	-.13*	-.07	-.10	-.35**
**Step 2: FFM Traits**											
Δ*R* ^*2*^	.30**	.22**	.24**	.24**	.30**	.12**	.06**	.15**	.32**	.18**	.18**
Neuroticism	-.14*	.08	-.11	.15*	.04	-.28**	-.12	-.41**	.05	-.41**	-.04
Extraversion	-.10	.04	-.09	-.14*	-.15*	.12	.21**	-.12	-.10	.13	-.30**
Agreeableness	-.53*	-.53*	**-.49**	-.40*	-.00	-.06	-.15*	-.08	-.50**	-.16*	-.25**
Conscientiousness	.01	.24*	.02	.14*	-.45**	.03	-.16*	.07	.01	-.04	.08

*Note.* FFM = Five Factor Model. ME = Machiavellian Egocentricity. RN = Rebellious Nonconformity. BE = Blame Externalization. CN = Carefree Nonplanfulness. SOI = Social Influence. F = Fearlessness. STI = Stress Immunity. SCI = Self-Centered Impulsivity. FD = Fearless Dominance. C = Coldheartedness.

** p < .01. *p < .05 Main effects > .30 indicated in **boldface**. ^a^F-change test significant at *p* < .05

In Step 2 in the American sample, FFM domains explained between 16 % (Fearlessness) and 46 % (Carefree Nonplanfulness) of the variance across the PPI-R scales and between 35 % (Coldheartedness) and 53 % (SCI) of the variance in factor scores. The FFM domains accounted for much less variance in the Egyptian sample, where the FFM domains explained between 6 % (Fearlessness) and 30 % (Carefree Nonplanfulness) of the variance across the PPI-R scales and between 18 % (Coldheartedness) and 32 % (SCI) of the variance in factor scores. With regard to specific associations between the FFM personality domains and PPI-R subscales, Machiavellian Egocentricity was related primarily to low Agreeableness in both samples; Rebellious Nonconformity was related primarily to low Agreeableness in both samples and to low Conscientiousness in the American sample; Blame Externalization was related to low Agreeableness in both samples and to Neuroticism in the American sample; Carefree Nonplanfulness was primarily related to low Conscientiousness in both samples; Social Influence was primarily related to Extraversion in the American sample but evidenced a significant negative association only with Neuroticism in the Egyptian sample; Fearlessness was most strongly associated with Extraversion in both samples; and Stress Immunity was primarily related to low Neuroticism in both samples.

Although all four FFM traits evidenced main effects in the explanation of SCI in the American sample, with the strongest associations deriving from low Agreeableness and low Conscientiousness, only low Agreeableness was uniquely associated in the Egyptian sample. Further, FD was explained by all four of the examined FFM domains in the American sample, with the strongest associations with low Neuroticism and Extraversion. In the Egyptian sample, however, FD was most strongly associated with low Neuroticism and was weakly associated with low Agreeableness; Extraversion was not uniquely associated with FD. Lastly, Coldheartedness was significantly explained by all four personality traits, with the strongest associations emerging for low Agreeableness and low Neuroticism. In the Egyptian sample, although low Agreeableness emerged as a significant predictor of Coldheartedness, the strongest predictor was low Extraversion. Examining the effects of Agreeableness and Conscientiousness, the domains that typically manifest the largest correlations with psychopathy scales [22] across the samples and psychopathy scores, Conscientiousness was a weaker correlate of psychopathy in the Egyptian sample (mean *β* = −.01) than in the American sample (mean *β* = −.24); the same was not true for Agreeableness, as the means were similar in the Egyptian (mean *β* = −.30) and American (mean *β* = −.37) samples.

With regard to the analyses examining moderation of associations by gender in Step 3, two significant interactions emerged in the American sample; the association between both Social Influence and Stress Immunity and Agreeableness was moderated by gender (*β*s = −.27 and -.21, respectively). Although Social Influence was largely unrelated to Agreeableness among males, it was strongly negatively associated among females. Further, the slope of the positive association between Stress Immunity and Agreeableness was steeper for males than for females. In the Egyptian sample, however, nine interactions emerged as significant in the explanation of the various PPI-R scales and factors. Agreeableness’ association with PPI-R Total Score, Machiavellian Egocentricity, Blame Externalization, and SCI were all significantly moderated by gender (*β*s = −.78, −.68, −.82, and -.77, respectively). Specifically, whereas these PPI-R scales/factors were largely unrelated to Agreeableness among male Egyptians, these scales/factors were negatively associated with Agreeableness among Egyptian women. Further, associations between both Carefree Nonplanfulness and Coldheartedness with Extraversion were also moderated by gender (*β*s = −.40 and .48, respectively). Whereas these associations were slightly/moderately negative among males, they were positive among females (and strongly positive for Carefree Nonplanfulness). Additionally, associations between Carefree Nonplanfulness and Stress Immunity with Neuroticism were also moderated by gender (*β*s = .45 and -.71, respectively). Specifically, Carefree Nonplanfulness was negatively associated with Neuroticism among males and strongly positively associated among females, and Stress Immunity was not associated with Neuroticism among men but was negatively associated among females. Lastly, the association between Coldheartedness and Conscientiousness was moderated by gender (*β* = .47). Whereas this association was largely nonexistent among males, Coldheartedness was positively associated with Conscientiousness among females. All told, gender appears to be much more important in the Egyptian sample than in the American sample in terms of accounting for the associations between psychopathy and FFM personality (the full set of results are available upon request from the first author).

### Factorial validity of PPI-R Psychopathy Dimensions

As described earlier, we next fit multi-group CFA models to examine the factorial validity of FD and SCI factors across the three samples. Model fit indices revealed a better fit for the model in which loadings were free to vary across groups (sample size adjusted BIC = 66572.52, AIC = 66485.00) when compared with the model in which loadings were constrained to be equal across groups (sample size adjusted BIC = 66732.05, AIC = 66654.95). This finding suggests a lack of factorial invariance of the PPI-R higher-order dimensions across cultures.

## Discussion

The current study is the first to examine the construct validity of self-reported psychopathy and its associations with basic FFM personality traits in the Arabic-speaking Middle East. Our results suggest that psychopathy appears broadly meaningful in the Arabic speaking Middle East, a culture markedly different from those in the Western world, where the vast majority of psychopathy research has been conducted. Specifically, as assessed by the PPI-R, psychopathy appears to demonstrate largely similar properties among American and Middle Eastern undergraduate samples Indeed, although internal consistencies across subscales were lower among the two Middle Eastern samples, scale interrelations were largely comparable, with the exception of Coldheartedness. Particularly, in the Saudi Arabian sample, the PPI-R Coldheartedness scale appeared to be considerably less associated with other features of psychopathy, suggesting that it may possess differing implications across cultures. This exception is important, however, given that some authors (e.g., [25]) regard a lack of capacity for deep love and a lack of capacity for guilt, both of which are assessed by the PPI-R Coldheartedness scale, to be among the core features of psychopathy. Further, again with some notable exceptions (i.e., Coldheartedness), associations between psychopathy and FFM personality domains were largely consistent across cultures/nations. Our results bear important implications for both the assessment of psychopathy cross-culturally/nationally, and the conceptualization of psychopathy more broadly.

### Associations between psychopathy and personality across nations

Our findings from both cultures corroborate previous results confirming that psychopathy is a multifaceted construct, as we found that different psychopathy dimensions often evidenced markedly different patterns of associations with FFM domains. This broad finding parallels that in previous studies in Western cultures [21]. The FFM domains explained a larger percentage of variance in the American as compared to the Egyptian sample, a finding that may be partly attributable to the higher reliability of these scores in the American sample. Nevertheless, across both samples, psychopathy was, with notable exceptions, accounted for by a largely similar constellation of traits drawn from multiple FFM domains. At the higher-order level in both samples, FD was most strongly explained by low Neuroticism, whereas SCI was most strongly explained by low Agreeableness. With regard to divergent associations, FD was moderately associated with Extraversion both in our bivariate and multivariate analyses in the American sample but this association became nonsignificant in in the Egyptian sample in the multivariate analyses. Moreover, SCI was moderately associated with low Conscientiousness in the American sample but not in the Egyptian sample.

Although the consistent overall negative association between Neuroticism and FD in both samples is consistent with previous meta-analytic findings [26], the associations between FD and Extraversion suggest that Miller and Lynam’s contention that FD can be viewed as stable Extraversion [26] may not hold across cultures. Indeed, although FD was significantly associated with Extraversion in both samples at the bivariate level, the magnitude of this association was significantly weaker in the Egyptian sample. Nevertheless, consistent with Miller and Lynam’s [26] meta-analysis, low Neuroticism consistently emerged as the personality domain most strongly associated with FD in both samples. When associations between FFM personality and individual subscales that load on FD were examined, a more nuanced picture emerged. Whereas Extraversion was associated with Fearlessness at the same magnitude in both samples, it was significantly more strongly related to Social Influence and Stress Immunity in the American sample, and essentially unrelated to Stress Immunity in the Egyptian sample. Nevertheless, it is important to note that some have contended that FD may play at most a secondary role in psychopathy [26], although others disagree [20].

Consistent with meta-analytic findings [26] and expectations, low Agreeableness emerged as strongly associated with SCI in both samples. Low Conscientiousness, however, evidenced a significant main effect on SCI in the American sample, yet was essentially unrelated in the Egyptian sample, consistent with a general pattern in which Conscientiousness was more weakly related to the PPI-R scales and factors in the multivariate analyses in the Egyptian sample. Again, an examination of associations between FFM traits and SCI revealed a more nuanced picture. Although Conscientiousness was similarly negatively associated with Carefree Nonplanfulness in both samples, associations with other subscales comprising SCI varied between samples. Indeed, whereas Conscientiousness was moderately negatively associated with Rebellious Nonconformity, Blame Externalization, and Machiavellian Egocentricity in the American sample, these associations were smaller in magnitude, or approached zero (for Machiavellian Egocentricity) in the Egyptian sample.

Notably, Coldheartedness evidenced more marked divergent associations with FFM personality traits across samples; similarly, it manifested divergent relations with the other PPI-R subscales across the two samples. Specifically, although Coldheartedness was most strongly associated with low Agreeableness, followed by a moderate association with low Neuroticism in the American sample, this dimension evidenced moderate associations with low Extraversion and low Agreeableness in the Egyptian sample. Taken together, it appears that in the American sample, Coldheartedness primarily represents a combination of low Agreebleness and low Neuroticism. In contrast, in the Egyptian sample, Coldheartedness represents a combination of low Agreeableness and low Extraversion. It is not clear from the current findings whether these divergent patterns of associations reflect problems with the Arabic version of PPI-R Coldheartedness, a true difference in the Coldheartedness construct, or both. That caveat notwithstanding, the current results suggest that the PPI-R Coldheartedness domain functions quite differently across Western and Middle-Eastern samples. These findings underscore the need for further investigation of the correlates of these dimensions across these two broad geographical and cultural groups.

As there are exceedingly few published data on Egyptian versus non-Egyptian differences in the correlates of personality, these findings are challenging to interpret. Ibraham [15] speculated that Extraversion may be manifested in more agentic behaviours (e.g., assertiveness, liveliness) in Western culture but more communal behaviors (e.g., social closeness, intimacy) in Egyptian culture [15], a conjecture that dovetails broadly with our findings. Nevertheless, our correlational differences will require replication, ideally with additional measures, in other cross-cultural samples.

With regard to gender, our results within the American sample were largely consistent with previous findings suggesting broadly comparable correlates of psychopathy across genders [7, 27]. Indeed, in this sample, few associations with FFM personality domains varied by gender across PPI-R scale and factor scores. In contrast, associations between various aspects of PPI-R psychopathy and FFM domains varied greatly in the Egyptian sample, with associations varying by gender for at least one of the FFM domains across 9 out of 11 of PPI-R scales and factors. Although preliminary and requiring replication, these results suggest that in contrast to Western samples, associations between psychopathy and FFM personality may vary substantially by gender in Middle-Eastern samples. Although we found differential reliabilities of PPI-R and NEO-FFI scales for men and women, the reliabilities were largely consistent for those associations that were most variable across genders, suggesting that these results are unlikely to be related to gender differences in item or scale functioning. Hence, our findings indicate that further investigation of gender differences in the correlates of psychopathy in Arabic cultures is warranted. Further, examination of the PPI-R validity scales suggested consistent responding to items. This finding addresses two other potential explanations for these differences, namely that (a) many of the psychopathy items were not translated appropriately or (b) participants experienced difficulty with understanding the meaning of many items. At the same time, the possibility that some of the items possess a different meaning across cultures, or that the construct of psychopathy varies across cultures and genders cannot be excluded.

We focused mainly on the external correlates of psychopathy measures across cultures. In a set of secondary analyses, we examined the factorial validity (internal structure) of these measures across cultures by fitting multi-group confirmatory factor analysis (CFA) models for FD and SCI to test the measurement equivalence of psychopathy higher-order dimensions across the three samples. Model fit indices revealed a better fit for the model in which loadings were freed to be vary across groups compared with the model in which loadings were constrained to be equal across groups. These results raise questions regarding how best to interpret our findings of cross-cultural differences for FD and SCI, as these differences may reflect differences in factor structure rather than true construct-level differences. These subsidiary analyses should be interpreted with caution, however, given both the modest sample sizes across groups as well as previous findings of largely similar factor structures of a different psychopathy instrument, the Psychopathy Checklist, Screening Version (PCL:SV; [14]) in Middle Eastern samples [39]. It will be therefore important for future research to more fully explore cross-cultural differences in psychopathy factor structure.

### Limitations

Our findings should be interpreted in view of several limitations. The use of undergraduate samples probably resulted in a smaller range for both predictor and criterion variables compared with forensic samples, potentially attenuating the magnitude of associations among variables. Further, although correlations corrected for attenuation were examined in bivariate analyses with FFM personality, the relatively low internal consistency of scales (particularly FFM scales) in the Egyptian sample may have attenuated the magnitude of the bivariate and multivariate associations. This may be particularly true in the context of the multivariate regression analyses, which may be reflected in the smaller percentage of variance explained by FFM personality domains across PPI-R scales and factors in the Egyptian sample. In addition, although not entirely inconsistent with previous findings failing to identify a clear-cut Openness dimension in non-Western samples (e.g., [37]), the low internal consistency of Openness in our Egyptian sample suggests that the development of more culturally-sensitive measures of Openness may be warranted. Indeed, in Arabic samples, Openness may assume a different form given that many of the items typically used to assess Openness may be less relevant in non-Western samples. The issue of low internal consistencies in Arabic samples is an important question worth investigating in its own right, particularly given the extremely low levels of deviant and inconsistent responding on the PPI-R validity scales. Indeed, items on certain personality scales may evidence lower internal consistencies in the Arabic samples for substantive reasons, including differences in internal structure. Finally, it will be important to ascertain that our overall findings hold across alternative translations of both psychopathy and FFM measures.

## Conclusions

All told, our results provide preliminary support for the cross-cultural/national generalizability of many of the core features of psychopathy and provide the first evidence of FFM-psychopathy correlates in a non-Western, Arabic-speaking sample. Consistent with findings for the FFM and other broad models of personality [23], the psychopathy construct appears to be meaningful in non-Western cultures, although it may differ in several important ways in such cultures. This conclusion underscores the cross-cultural importance of the psychopathy construct and should encourage future research in Middle Eastern populations. Moreover, we also found provisional evidence for substantial gender differences in the relations between self-reported psychopathy and personality in a Middle-Eastern country (Egypt) but not in the United States, suggesting that additional research on the intersection between gender differences and culture in the psychopathy domain is warranted. Our findings underscore the point that further research on cross-cultural differences in personality disorders broadly, and psychopathy more specifically, has the potential to inform both clinical assessment and treatment.

## Data availability

All data are available upon request from the first author (RDL).
