# Erratum to: self-reported psychopathy in the Middle East: a cross-national comparison across Egypt, Saudi Arabia, and the United States

**DOI:** 10.1186/s40359-015-0098-8

**Published:** 2015-11-24

**Authors:** Robert D. Latzman, Ahmed M. Megreya, Lisa K. Hecht, Joshua D. Miller, D. Anne Winiarski, Scott O. Lilienfeld

**Affiliations:** Department of Psychology, Georgia State University, Atlanta, 30302-5010 GA USA; Department of Psychological Sciences, Qatar University, Doha, Qatar; Department of Psychology, University of Georgia, Athens, GA USA; Department of Psychology, Emory University, Atlanta, GA USA

Erratum

After publication of this article [1], it was noticed that Ahmed M. Megreya’s name had been misspelt and was incorrectly presented as Ahmed M. Megraya. The correct spelling of the author’s name, Ahmed M. Megreya, is included in the author list above and has been updated in the original article [1].
